# Supplementary material for: Zuccagnia punctata Cav., a Potential Environmentally Friendly and Sustainable Bionematicide for the Control of Argentinean Horticultural Crops
Source: Plants (Basel). 2023 Dec 7;12(24):4104. doi: 10.3390/plants12244104 (PMC10747203; doi:10.3390/plants12244104)
Supplement: Supplementary file 1 [file plants-12-04104-s001.zip › plants-2695807-supplementary.pdf]

Supplementary Materials

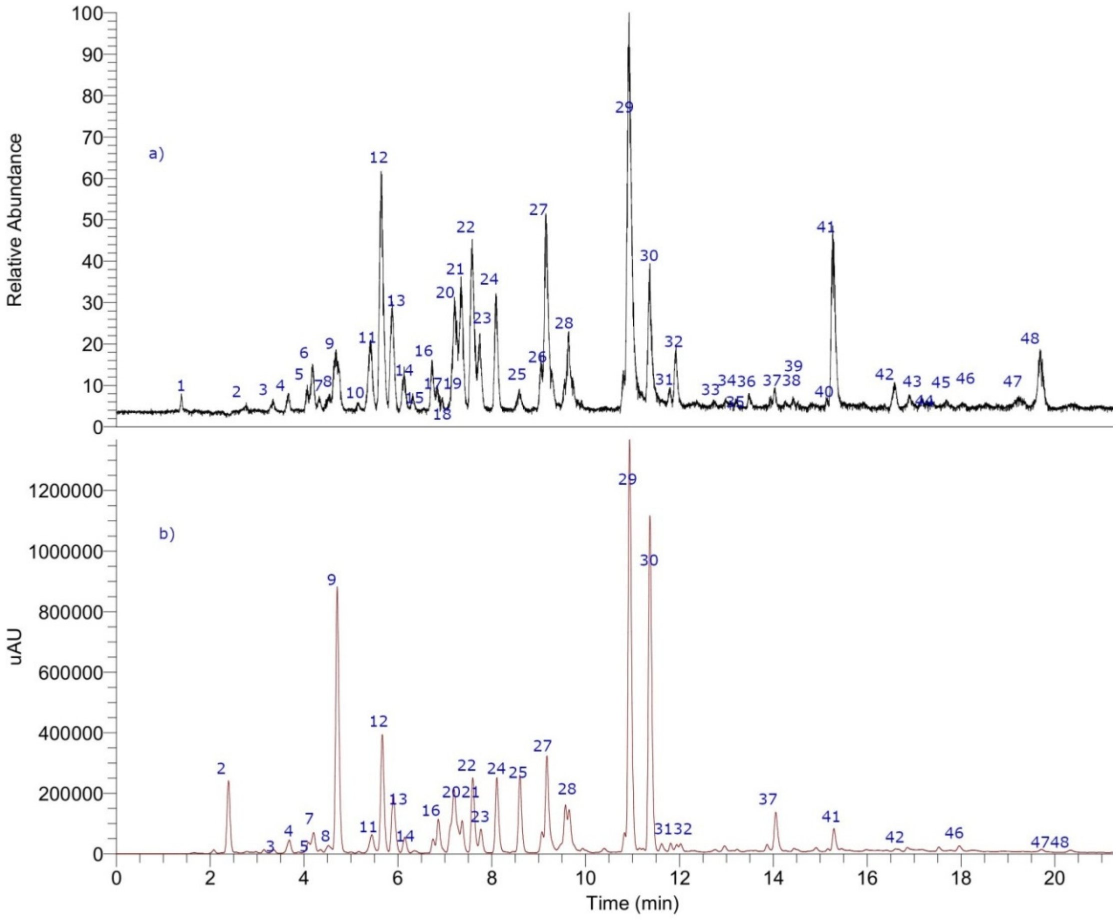

Figure S1. The HPLC-MS Fingerprints of ZpRe resin: (**a**) The total Ion Current (TIC) chromatogram and (**b**) the UV-vis chromatogram at 280 nm.

Table S1. High resolution UHPLC–PDA–Q-Orbitrap identification of biomolecules from ZpRe resin.

	**Retention Time (min.)**	**UV Max**	**Tentative Identification**	**Elemental Composition [M–H]⁻**	**Theoretical Mass (*m*/*z*)**	**Measured Mass (*m*/*z*)**	**Accuracy (δ ppm)**	**MS^n Ions**
1	1.33		unknown	$C_{16}H_{15}O_5$	85.00343			
2	2.77	279	Naringenin	$C_{15}H_{11}O_5$	271.06110	271.0601	3.67	

3	3.35	279-367	Shikoniin	$C_{16}H_{15}O_5$	287.09325	287.0923	3.40	
4	3.65	279	Afzelechin	$C_{15}H_{13}O_5$	273.07575	273.0766	3.22	
5	4.05	-	Calonectrin	$C_{19}H_{25}O_6$	349.16456	349.1654	2.58	85.00342
6	4.18	279	EpiAfzelechin	$C_{15}H_{13}O_5$	273.07575	273.0766	3.10	
7	4.28	287	Naringenin isomer (could be butein)	$C_{15}H_{11}O_5$	271.06195	271.0611	3.67	151.0394: 109.0286
8	4.31	287	3,7- dihydroxiflavanone	$C_{15}H_{11}O_4$	255.06519	255.0661	3.67	151.0394: 109. 0286
9	4.61	287	7,8-dihydroxiflavone	$C_{15}H_{11}O_4$	255.06519	255.0661	3.11	237.0553;
10	5.12	287	5-Hydroxy-4',7-dimethoxyflavanone	$C_{17}H_{15}O_5$	299.09140	299.0929	2.95	285.0403; 179.0345, 135.0444 (100)
11	5.38	287	3,7,8 Trihydroxydihydroflavanone	$C_{15}H_{11}O_5$	271.06010	271.0610	3.33	253.0503; 225.0552; 197.0603; 151.0029
12	5.67	234-292-[illegible]25	1-methyl-3-(3',4'-dihydroxyphenyl)-propyl caffeicacid ester	$C_{19}H_{19}O_6$	343.11761	343.1187	2.49	179.0344; 161.0236; 135.0443
13	5.88	236-277-[illegible]12	1-methyl-3-(3',4'-dihydroxyphenyl)-propyl caffeic acid ester isomer	$C_{19}H_{19}O_6$	343.11761	343.1183	2.22	257.0818; 179.0345; 151.0393; 135.0444; 107.0494
14	5.88	236-277-[illegible]12	Guibourtinidol	$C_{15}H_{13}O_4$	257.08084	257.0816	3.10	179.0345; 151.0393; 135.0444; 107.0494
15	6.14	279-367	1-methyl-3-(3',4'-dihydroxyphenil)-propyl caffeic acid ester isomer	$C_{19}H_{19}O_6$	343.11853	343.1187	2.66	287.0818; 151.0393; 119.0495; 107.0494
16	6.14	279-367	Shikoniinisomer	$C_{16}H_{15}O_5$	287.09140	287.0923	3.18	151.0393; 119.0495; 107.0494
17	6.27	285	7,4'-dihydroxy-5methoxy flavanone	$C_{16}H_{13}O_5$	285.07575	285.0766	3.19	149.9952; 119.0495
18	6.73	287	Dihydroxyflavanone	$C_{15}H_{11}O_4$	255.06519	255.0669	3.05	237.0553; 209.0604; 195.0400

19	6.91	251-349	Rhamnetin	$C_{16}H_{11}O_7$	315.04993	315. 0511	3.18	185.00346, 146.93796
20	7.18	246-324	3,7-dihydroxyflavone	$C_{15}H_9O_4$	253.05029		2.99	208.0524; 223.0326
21	7.31	277 314	1-methyl-3-(4'-hydroxyphenil)-propyl caffeic acid ester	$C_{19}H_{19}O_5$	327.12357	327.1227	2.64	135.0443;
22	7.55	242, 291-324	2-methyl-3-(3-hydroxy-4'-methoxyphenyl)-propyl caffeic acid ester	$C_{20}H_{21}O_6$	357.13409	357.1332	2.32	343.1104; 193.0500; 179.0343; 161.0237; 135.0440;
23	7.70	249-285-323	1-methyl-3-(4'-hydroxyphenil)-propyl caffeic acid ester isomer	$C_{19}H_{19}O_5$	327.12380	327.1227	3.01	179.0344; 163.0394; 135.0443; 119.0494
24	8.04	235-343	Pinocembrin(5, 7-dihydroxyflavanone)	$C_{15}H_{11}O_4$	255.06601	255.0651	3.23	227.0907; 213.0503; 164.0109; 151.0029; 123.0080
25	8.54	239-306	2´hidroxy-4methoxichalcone	$C_{16}H_{13}O_3$	253.08592	253.0866	3.02	
26	9.00	291	Pinocembrinisomer	$C_{15}H_{11}O_4$	255.06599	255.0651	3.17	227.0709; 213.0553; 164.0109; 145.0642; 123.0080
27	9.14	267 315 360	Galangin(3,5,7-trihydroxyflavone)	$C_{15}H_9O_5$	269.04579	269.0453	3.22	213.0551; 169.0653;
28	9.61	242-268-310-357	Caffeic acid phenetylesther	$C_{17}H_{15}O_{14}$	283.09649	283.0794	3.38	
29	10.90	232-346	2',4'-dihydroxychalcone	$C_{15}H_{11}O_3$	239.07027	239.0710	2.91	197.0603; 169.0653; 153.0186; 135.0080;
30	11.37	232-345	2',4'-dihydroxy-3'-methoxychalcone	$C_{16}H_{13}O_4$	269.08167	269.0808	3.08	
31	11.88	231-308-347	1-methyl-3-(4'-hydroxyphenyl)-propyl p-coumaric acid ester isomer	$C_{19}H_{19}O_4$	311.12866	311.1289	2.81	179.0344; 163.0394; 135.0444; 119.0494
32	12.01	277-312	Dunnione	$C_{15}H_{13}O_3$	241.08592	241.0866	2.98	
33	12.78	287	Flavanone	$C_{15}H_{11}O_3$	239.07027	239.0709	2.91	197.0603; 169.0653; 153.0186;

								135.0080; 121.0280
34	13.00		unknown					
35	13.37		unknown					
36	13.50	285	Blestriarene B	$C_{30}H_{23}O_6$	479.14957	479.14891	1.36	
37	14.10	280-323	4'-terbutyloxyphenyl p-coumaricacidesterisomer	$C_{19}H_{19}O_4$	311.12779	311.1286	2.71	179.0344; 161.0237; 135.0442;
38	14.52	283	Glyvenol	$C_{29}H_{33}O_6$	477.22754	477.2271	0.78	
39	14.55	293	1-methyl-3-(3',4'-dihydroxyphenil)-propyl caffeic acid ester isomer	$C_{19}H_{19}O_6$	343.11761	343.1183		179.0344; 161.0238;135.0442; 109.0286
40	15.40	280	Vedelianin	$C_{29}H_{35}O_6$	479.24882	479.2433	1.17	
41	15.47	280	Hidroxivedelianin	$C_{29}H_{35}O_7$	495.23773	495.2381	0.82	161.0238; 135.0443; 109.0286
42	16.60		3,7-dimethyl-2,-octaenyl caffeic acid ester	$C_{19}H_{21}O_4$	313.14344	313.1443	2.91	
43	17.04	267-357	Hidroxivedelianinisomer	$C_{29}H_{35}O_7$	495.23785	495.2377	0.25	479.24323, 239.0710; 179.0345; 161.0238; 135.0442;
44	17.12	285	Vedelianinderivative	$C_{29}H_{33}O_6$	477.22717	477.2276	1.10	
45	17.56	285-320	3,7-dimethyl-2,6-octadienyl caffeic acid ester (geranyl Caffeate)	$C_{19}H_{23}O_4$	315.16993	315.1600	3.10	178.0265; 134.0364; 133. 0289
46	17.98	289-320	Lupinifolin	$C_{25}H_{25}O_6$	405.16965	405.1754	-1.27	
47	19.58	289	Vedelianinisomer	$C_{29}H_{35}O_6$	479.24882	479.2433	1.17	
48	19.99	287	Shinflavanone	$C_{25}H_{25}O_4$	389.17474	389.1756	2.37	371.1654
